# The Implementation of a Caregiver Distress Screening Initiative in a Pediatric Oncology Healthcare Setting: A Quality Improvement Project

**DOI:** 10.3390/curroncol33050252

**Published:** 2026-04-28

**Authors:** Clayton Culp, Christen Long, Angelique Ribieras, Erica H. Sirrine

**Affiliations:** Department of Social Work, St. Jude Children’s Research Hospital, Memphis, TN 38105, USA; christen.long@stjude.org (C.L.); erica.sirrine@stjude.org (E.H.S.)

**Keywords:** distress screening, caregiver support, psychosocial support, psychosocial care, psychosocial oncology, pediatric oncology, childhood cancer, oncology social work

## Abstract

**Background**: Parents of children with cancer often experience significant stressors that can heighten distress and impact overall well-being. Despite recommendations supporting distress screening for adult and pediatric patients with cancer, systematic assessment of parent or caregiver distress in pediatric oncology settings remains limited. This Quality Improvement initiative aimed to implement routine distress screening for adult caregivers of pediatric patients with brain and solid tumors at our institution. **Methods**: Using the 18-item CancerSupportSource™-Caregiver (CSS-CG) instrument, our social work team invited eligible caregivers to complete monthly electronic distress screeners while their child was receiving treatment. Automated reports with tailored psychoeducational resources were sent to caregivers upon completion, and social workers received alerts when participants scored at-risk for anxiety, depression, or financial strain. The feasibility of the CSS-CG implementation was assessed via caregiver enrollment and completion rates, and clinician acceptability was evaluated through a post-implementation survey. **Results**: Of 122 eligible patients, 64 of their caregivers enrolled in the CSS-CG screening platform (52.45%), and 44 (36.06%) completed at least one screener. Of the 44 caregivers who completed the CSS-CG assessment, 59.09% (*n* = 26) screened positive at least once for risk of anxiety, 54.54% (*n* = 24) for risk of financial strain, and 59.09% (*n* = 26) for risk of depression. Among participating social workers, 83% reported that the screener improved their clinical practice, making their sessions more focused and helpful. They also noted that it improved their ability to identify a caregiver’s overall needs. **Conclusions**: Implementing caregiver distress screening positively impacted social work practice at our institution. Despite variable caregiver enrollment and a few implementation challenges, the findings support the importance and potential benefit of screening for caregiver distress in a pediatric oncology setting.

## Simple Summary

Pediatric cancer impacts the entire family system, especially a child’s parent or primary adult caregiver. Caregivers are often forced to juggle multiple demands while a child is in treatment, leading to significant challenges, including practical, emotional, social, and financial stressors. Distress screening for pediatric patients is not always routine, and screening for their adult caregivers is even more limited. This Quality Improvement Project aimed to implement routine distress screening for caregivers of patients with brain and solid tumors at our institution. Findings from this project highlight the potential benefits and challenges of such screening and can provide a foundation for other pediatric oncology psychosocial departments considering the implementation of standardized caregiver distress screening.

## 1. Introduction

Pediatric illness affects not only the diagnosed child but also the entire family [1,2,3,4]. Parents or other adult caregivers of children with serious illnesses, including cancer, face multiple stressors, including the pressures of making high-stakes medical decisions, the emotional impacts of having an ill child, relocation or travel for medical care, and financial strain [5]. These challenges usually accompany a parent’s need to manage competing responsibilities, including work and the care needs of other family members [6]. Research indicates that parents of children with chronic illnesses experience increased anxiety, depression, and post-traumatic stress symptoms when compared to parents of children without chronic illnesses [7,8,9,10].

While there is evidence for parental resilience in the face of such stressors [11,12,13,14], the psychosocial toll of pediatric cancer on parents can be immense [7]. This is often described as “distress,” a term defined by the National Comprehensive Cancer Network as “a multifactorial unpleasant experience of a psychological (i.e., cognitive, behavioral, emotional), social, spiritual, and/or physical nature that may interfere with the ability to cope effectively with cancer, its physical symptoms, and its treatment. Distress extends along a continuum, ranging from common normal feelings of vulnerability, sadness, and fears to problems that can become disabling, such as depression, anxiety, panic, social isolation, and existential and spiritual crisis” [15] (p. 6).

For parents of children with serious illnesses, including cancer, distress can sometimes present as depression, anxiety, post-traumatic stress symptoms, or feelings of loneliness and isolation [16,17,18]. Parental distress has also been associated with greater patient distress [19], and poorer health-related quality of life for the patient in treatment [20,21,22]. Research suggests that higher levels of distress and poorer coping may be predictive of later difficulties [17], so early identification of caregiver distress can give clinicians the opportunity to intervene more proactively, providing psychosocial support and resources to promote or enhance individual and family resilience.

Distress screening in oncology has garnered increased attention in recent years [9,23,24,25]. However, the focus has often been on distress screening for patients as highlighted by the American College of Surgeons Commission on Cancer’s (CoC) requirement that accredited adult oncology centers conduct routine psychosocial distress screening [26]. While both adult and pediatric centers recognize the burden of caring for someone with a cancer diagnosis, distress screening for caregivers of patients is not always routine [23,25]. In 2015, the Standards for the Psychosocial Care of Children with Cancer and their Families were developed to provide pediatric oncology institutions with guidance on best practices for providing psychosocial care. The Standards include many recommendations for routine assessment and screening, including considerations for patient and family mental health and coping, as well as the financial hardships often associated with a cancer diagnosis [12,27,28]. A recent survey of 129 Children’s Oncology Group (COG) programs revealed that many institutions rely on informal conversations with patients and families rather than using validated psychosocial assessment or distress screening tools on a routine basis [29]. Without systematic procedures in place, it can be challenging for social workers and other psychosocial clinicians to prioritize the ongoing assessment of caregiver distress.

Several studies have explored the implementation of parental distress screening, and the existing literature suggests that both parents and social workers find it to be beneficial and acceptable [21,30,31]. Notably, a qualitative study of key stakeholders, including multidisciplinary health care providers and parent advocates, highlighted the importance of framing screening as an opportunity for more “efficient and effective” social work practice [32] (p. 5). Unfortunately, many pediatric oncology social workers face high caseloads, emotional stress, and documentation burdens, so it is essential that screening processes meaningfully enhance social work practice without being overly burdensome for clinicians.

With these challenges in mind, our social work department prioritized distress screening in our 5-year strategic plan. To meet this aim, we conducted a quality improvement (QI) project to implement parental distress screening as part of routine social work practice for pediatric patients diagnosed with brain and solid tumors. Our department is fortunate to have staffing that enables all patients and families to receive initial and ongoing social work assessment and support throughout the treatment trajectory as a universal psychosocial intervention, aligned with Kazak’s three-tiered model of psychosocial support [8,33]. For this reason, while distress screening is often used to triage the provision of social work services or interventions with patients and families, this project aimed to enhance the psychosocial care already being provided by helping social workers better detect unmet parental needs and provide more collaborative, intentional, focused, and meaningful sessions.

## 2. Materials and Methods

A working group of six social workers within our department was created to conduct a literature review of available distress screening tools, investigate best-practice recommendations, and gather information and feedback from peer institutions engaged in distress screening. Additionally, the working group surveyed other social workers within the department and the parents of our pediatric patients to identify common concerns that would be most helpful to address as part of the distress screening process (e.g., financial, emotional, etc.). The working group’s findings were compiled into an internal report that informed subsequent decisions about which distress screening tool to use and how to implement it in the pilot project. Two social workers were identified to serve as leaders for the QI initiative and were responsible for developing and conceptualizing the project, with consultation from the department director. The project leaders developed a detailed protocol and trained their social work colleagues to implement the distress screening program alongside them.

### 2.1. Instrument Selection

Since the primary aim of the QI project was to improve the focus and quality of social work interventions, the initial plan was to have social workers administer in-person, paper-based screeners at the start of a family’s social work visit. Several instruments were considered by the team, and the CancerSupportSource^®^ Caregiver also known as the CSS-CG (Washington, DC, USA) was ultimately selected for use, in part because of its electronic administration via an external online form (MyCareReport). Numerous studies point to the benefits of electronic screening, as it can enable easier analysis of electronic data to assess program performance and guide practice improvements [24]. While it is possible to integrate the CSS-CG into the electronic health record (EHR), our team chose to keep it separate for the pilot project due to privacy concerns regarding storing personal parent distress information in pediatric patient charts.

The CSS-CG is an 18-item measure using a 5-point Likert scale assessing caregiver concerns across five key domains: patient well-being, healthy lifestyle, caregiving tasks, finances, and emotional well-being. It includes one additional screening item to assess tobacco, alcohol, or other substance use. The CSS-CG has demonstrated strong internal consistency reliability (α = 0.92) and test-rest reliability (0.86), as well as acceptable convergent and discriminant validity [34]. Scales to flag respondents at-risk for clinical levels of depression (CSS-D2) and anxiety (CSS-A2) are embedded within the CSS-CG, with pre-defined cutoff scores demonstrating adequate sensitivity and specificity when compared with previously validated measures. The CSS-D2 flag is scored using the caregiver’s level of endorsement on two items, “Feeling sad or depressed” and “Feeling lonely or isolated,” and the CSS-A2 flag is scored using the items, “Worrying about the future and what lies ahead” and “Feeling nervous or afraid.” Additionally, the QI project leaders created a flag for financial distress that was triggered by any level of endorsement of the item “Managing household finances.”

### 2.2. Participants

Parents or other adults responsible for the care of pediatric patients at our institution are collectively referred to as “caregivers,” so we will use this term throughout the paper to describe any parent or adult caregiver who participated in the QI initiative. Although the hospital serves patients with many diagnoses through a variety of specialized clinics, the selected patient population was intentionally limited for this project. Since the two project leaders were already caring for patients with brain and solid tumors, those patients were selected exclusively for inclusion. This choice allowed the team to tailor the distress screening program to the specific needs of this patient population and effectively manage the administrative workload of overseeing the process.

Eligible caregivers included those of new patients (newly diagnosed and those previously treated elsewhere who were now seeking additional care at our institution) and existing patients restarting treatment due to disease recurrence. Caregivers of patients who were not receiving cancer-directed therapy (e.g., those followed for serial imaging only) and those receiving only a short course of therapy (e.g., six weeks of radiation) were excluded. Caregivers of adult children, who comprise only a small subset of patients, were also excluded, as the team believed the issue of privacy would be more difficult to navigate. While the CSS-CG was recently adapted for Spanish-speaking caregivers (CSS-Spanish), it was only available in English at the time of our QI initiative [35]. Therefore, caregivers were required to be proficient in English to participate.

### 2.3. Procedures

As previously mentioned, each patient and family at our institution is assigned to a social worker who conducts a comprehensive psychosocial assessment and follows the patient throughout their treatment trajectory to provide ongoing psychosocial assessment, support, and counseling. Between June 2024 and March 2025, adult caregivers of 122 patients diagnosed with brain and solid tumors met the eligibility criteria. Of the 122 eligible patients, the assigned social workers chose not to provide information about the screener to caregivers for 28 patients, based on clinical judgment that the screener or its timing would not be helpful.

Caregivers of the 94 remaining eligible patients were presented with information about the distress screening instrument and process during their initial or follow-up social work appointments. They were also informed that participation was completely voluntary and non-participation would not affect the standard social work services the patient or caregiver received. Caregivers of 64 eligible patients expressed interest in participating in the distress screening process. After providing verbal consent to participate, they were enrolled in the distress screening platform by their social worker. Enrollment consisted of entering basic information for the caregiver, including a phone number and/or email address, depending on their preferred method of survey completion (e.g., mobile device or website). Of the 64 patients whose caregivers enrolled, 36 had at least one of their adult caregivers complete the distress screening instrument at least once.

Caregivers received a monthly link sent by the platform (via text message or e-mail) to complete the screener at their own convenience for the duration of their child’s treatment. Upon completion of the screener, caregivers received an automated report (within the survey browser and via email) with psychoeducational resources tailored to their responses, ensuring that their identified concerns were promptly acknowledged and addressed, even before the social worker contacted them for follow-up. Prior to launching the distress screening initiative, our team tailored the report and accompanying resources to our specific patient population and institution. The social worker was also provided an automated clinical report upon caregiver completion that included “flags” for depression, anxiety, and/or financial needs when caregiver responses placed them at greater risk across these three domains. When one or more areas of concern were noted, the QI protocol required the social worker to contact the caregiver within 1–3 business days to address those specific needs and provide additional assessment and support. If no areas of concern were noted, the social worker used their clinical discretion to follow up within a timeline they deemed appropriate.

### 2.4. Implementation Outcomes

As noted earlier, our goal for the project was to more systematically assess caregiver distress while improving the quality of our social work sessions and interventions. To our knowledge, all prior use of the CSS-CG has been with adult caregivers of adult oncology patients, and we are the first pediatric institution to use the CSS-CG with adult caregivers of pediatric oncology patients.

The feasibility of using the CSS-CG was assessed through the caregiver enrollment rate and screener completion percentage. Caregivers were not surveyed regarding their experiences with the CSS-CG to avoid survey fatigue. After completion of the QI initiative, clinician acceptability was explored through a 21-item survey of participating social workers. The survey was internally developed by the leaders of the QI project and administered via Microsoft Forms (Redmond, Washington, USA). The survey included 18 items on a 5-point Likert scale assessing their overall satisfaction with the distress screening process, including its impact on their sessions with caregivers, its effectiveness in helping them better identify caregiver stressors, and the instrument’s feasibility and ease of administration. The survey also included the following open-ended questions to gather more in-depth feedback: (1) “Are there any other positive effects on your practice that you would like to share?” (2) “How has using this screening tool impacted your working relationship with the family?” and (3) “Are there any other challenges or suggestions you’d like to share?” Social workers were not anonymous in their completion to enable the team to openly discuss and follow up on any process and implementation concerns more effectively.

### 2.5. Data Analysis

This project was undertaken as a Quality Improvement (QI) initiative to improve internal processes and patient/family care, so a review by the Institutional Review Board (IRB) was not required, and written consent was waived. All social workers who participated in the follow-up survey provided written permission for their qualitative feedback to be shared in this article. Quotes were de-identified and reveal insights into our evaluation of the project at our institution. Demographic and frequency data were analyzed and reported using Microsoft Excel. Reporting of this QI project is consistent with the Standards for Quality Improvement Reporting Excellence (SQUIRE) 2.0 guidelines.

## 3. Results

### 3.1. Demographics

Of the 94 patients whose caregivers were eligible to enroll in the distress screening initiative and received information about the screener, 64 requested enrollment in the distress screening platform. Of those enrolled, 56% (*n* = 36) had one or more caregivers complete the distress screening instrument at least once. Eight of the 36 enrolled patients had two caregivers complete the screening at least one time, bringing the total number of enrolled caregivers to 44. Twenty-five (57%) of the enrolled caregivers completed at least half of the surveys sent to them, and 11 caregivers completed all of the surveys. Demographic results are shown in Table 1.

### 3.2. Caregiver Distress

Of the 44 caregivers who completed the CSS-CG, 59.09% (*n* = 26) screened positive at least once for risk of anxiety, 54.54% (*n* = 24) for risk of financial strain, and 59.09% (*n* = 26) for risk of depression. Thirteen caregivers (29.55%) screened positive for all three of these risk factors at least once, whereas only six caregivers (13.63%) scored negative for all three risk factors each time the instrument was completed.

Caregivers endorsed experiencing a variety of concerns. Two items were endorsed by more than 50% of participants as moderate, serious, or very serious concerns based on average item scores: “worrying about the future and what lies ahead” (61%) and “disruptions in work, school, or home life” (57%). The items that at least 25% of caregivers identified as moderately, seriously, or very seriously concerning to them are listed in Table 2.

### 3.3. Social Worker Response to the Distress Screening Process

Eight social workers in the department with state licensure (Licensed Master Social Worker or a Licensed Clinical Social Worker) and one Master of Social Work (MSW) student intern had multiple caregivers eligible to participate in the QI initiative. Two additional social workers had only one eligible caregiver assigned to them.

Caregiver enrollment rates varied by social worker, ranging from 25% to 72%, with an average of 47.63%. Following completion of the QI project, six social workers completed the 21-item survey. To ensure objectivity, the two social workers co-leading the distress screening initiative did not complete the survey. In addition, the two social workers who had only one eligible caregiver and the MSW intern did not complete the survey because of their limited experience with the distress screening process. Quantitative results of this survey are shown in Table 3.

Eighty-three percent of the six social workers surveyed reported that using the distress screener was worth their time and effort because it improved their clinical practice. They reported that the screener made their sessions more focused and helpful. Sixty-seven percent agreed that their sessions were more meaningful or intentional because of the distress screener, and 50% reported their sessions were more collaborative. One social worker stated, “*My introduction of the tool to the caregiver helps reinforce the idea that my role is primarily for caregiver support. Even if they do not engage with the screener, I have communicated that I am here for them and want to be engaged with their areas of distress.*” Another expressed, “*I have been able to be more intentional with my sessions and address concerns that the caregivers may not be ready to verbalize without a prompted question.*”

Eighty-three percent of social workers agreed or strongly agreed that using the distress screener helped them better identify a caregiver’s overall needs. Every respondent reported that it helped them better identify concerns about a caregiver’s ability to manage life outside of the hospital, and 83% noted that it helped them identify concerns about managing their child’s life and care at the hospital. Eighty-three percent also indicated that the screener helped them recognize caregiver concerns. These concerns did not necessarily lead directly to referrals, as only one social worker endorsed frequently or very frequently making a referral they would not have otherwise made without the information obtained from the screener. One social worker stated, “*For those caregivers who may be more forthcoming via survey than in person, this has helped me gain greater insight into concerns and stressors our caregivers are experiencing, as well as a tool to use to foster meaningful conversations and outcomes.*” Another shared, “*The results of the distress screener helped to identify worries that families sometimes will not express verbally… It has been a great enhancement to my practice.*”

Eighty-three percent of social workers agreed that the instrument was easy to incorporate into their practice, the amount of time they spent administering the tool was reasonable, and that using the distress screener positively impacted their practice. However, only 33% reported that caregivers were willing to complete the instrument and were unsure whether caregivers found the screening tool beneficial. One respondent stated, “*The screener did open up some conversation topics, but I did not have consistent participation from families. Most times I was aware of the distress without the screener.*” Another explained, *“…Also, caregiver follow-through on the screeners is hit-or-miss—I have not brought it up later in future sessions to remind them to fill them out, because I do not want to add burden/shame for not doing it, but perhaps I should remind them. Generally, just remembering to add it into my routine practice during introductory visits was challenging, but over time it has become more natural.*” In follow-up discussions with the social workers who participated in the project, similar challenges were identified, including that survey fatigue or survey confusion (e.g., patients/caregivers receiving frequent or similar surveys from various healthcare institutions) could negatively impact caregiver participation. In addition, they noted that using a third-party platform (e.g., CSS-CG MyCareReport) to administer the instrument could deter some caregivers from participating. This was also evident in the social worker survey, where one respondent stated, “*I understand why, but I think having the screener in a separate website may have deterred some caregivers from completing consistently and required additional mental load from providers.*”

## 4. Discussion

The stress of pediatric illness, especially cancer, impacts the entire family system. This QI initiative aimed to more systematically assess the distress of primary caregiver(s) to enhance the psychosocial care provided by our social work team. We hoped the distress screener would help social workers more easily identify parental concerns and provide targeted interventions to address any significant areas of need.

Overall, the results of our post-survey indicated that social workers were satisfied with the distress screening instrument and process. There was strong agreement that using the screener made their work with families more focused and helpful while also improving their ability to identify the needs of our caregivers. Multiple social workers reported a belief that some caregivers endorsed concerns that they may have been unwilling or uncomfortable to express verbally during a social work session via the screener. Moreover, social workers stated that it helped clarify their role to caregivers as one that includes supporting their mental health concerns and needs.

Since pediatric oncology social workers often face competing demands on their time, we were pleased to learn that most of the team reported that the distress screener was easy to integrate into their existing practice and workflow. However, the social workers also identified a few challenges. Although all but one social worker felt the distress screening process was worth their time and effort, the team was unsure whether the caregivers would be willing to complete the screeners or find the survey beneficial. It’s possible these perceptions influenced how social workers introduced the screener’s purpose and benefits to caregivers.

There were also notable differences between social workers in caregiver enrollment rates, ranging from 25% to 72%. This could be due to variations in how and when social workers offered the screener to caregivers. We were initially concerned that caregiver enrollment and completion rates were relatively low. However, since the caregiver is not our patient and therefore not required to participate in social work services or intervention, an enrollment rate of 52.45% and a completion rate of 56% were acceptable. In addition, this was the first time our department has implemented a caregiver screening instrument, so these rates may improve in the future as we incorporate the insights gained from this QI project. Some of these difficulties and intragroup differences were expected as a normal part of implementing a change in practice. Further exploration is needed to understand these differences, but it suggests that the enrollment percentage could be improved with additional training and support for social workers.

Although the main purpose of this QI project was to impact social work practice rather than draw generalizable conclusions about caregiver distress at our institution, the descriptive data from the surveys offer insights for other psychosocial teams considering the implementation of routine caregiver distress screening at their hospitals. Consistent with other publications highlighting the impact of illness on caregiver mental health and well-being [7,8,9,10], the results of our project also reflected a population of caregivers experiencing significant levels of distress, with most screening positive at least once for depression, anxiety, and financial strain. Anxiety was a particular concern, with two of the top five most strongly endorsed concerns related to worry, nervousness, or fear. This finding highlights the need for our social work team to pay particular attention to assessing and providing psychoeducation on common caregiver worries and fears, even among caregivers who do not complete a formal distress screener. A focus on caregiver mental health is also consistent with a recent call to action to address caregiver mental health needs in pediatric oncology [9].

While over half of the participants endorsed moderate to serious concerns about depression, anxiety, or financial strain, a small percentage (13.63%) of caregivers did not report significant levels of distress in any of these areas. This finding is consistent with other research on resilience, which suggests that although initial distress is common, most caregivers of children with serious illness demonstrate resilience over time [14,36,37]. For this reason, our department’s new strategic plan aims to explore ways to more systematically incorporate patient, caregiver, and family interventions and psychoeducation focused on building resilience.

As we consider expanding caregiver distress screening to other patient diagnoses within our institution, future exploration will include seeking input from caregiver participants about their experience with the process. Future QI studies in this area could also include data on caregivers’ perceptions of distress screening, including perceived burdens and benefits of participation. Such insight could help alleviate concerns among social workers who fear that participation might be too burdensome for caregivers or that caregivers might not find it useful, a concern that has been noted elsewhere [21,38].

### Limitations

Despite our meaningful findings, several important limitations should be noted. This project was conducted solely for quality improvement purposes, so the results are not generalizable beyond our institution. Our findings may have also been influenced by the specific patient population selected, our institutional staffing resources, and our departmental workflows.

In addition, the sample size for our social work team piloting the project was very small, and the post-survey was developed by the staff leading the initiative and was not a previously validated measure. While our team is intentional about engaging in open, honest conversations about internal practices and processes, the social worker survey was not anonymous, which may have led to social desirability bias or discouraged critical feedback. We also only assessed social workers’ perceptions of caregiver benefit. We did not solicit caregiver perceptions of the process or the benefits of their participation, which further limits the generalizability and feasibility of this quality improvement project.

Finally, at the time of our project, the CSS-CG tool was only available in English, so speakers of other languages could not participate. It is also unclear whether caregivers who chose to participate in the distress screening QI initiative had levels of distress similar to those who declined to participate, further indicating that the results should not be generalized beyond this project. Selection bias should also be considered, as participating social workers did not provide screener information to 28 of the 122 eligible patients and their caregivers based on their clinical judgment that the screener would not be helpful or the timing was not appropriate given the unique patient or family circumstances.

## 5. Conclusions

While distress screeners often focus on measuring the stressors of adult or pediatric oncology patients, assessing the level of perceived distress among their primary caregivers can help social workers tailor interventions to best meet the psychosocial and support needs of a patient’s caregiver and family. This QI project aimed to systematically screen for distress in caregivers of pediatric oncology patients at our institution. Despite some challenges with caregiver enrollment and completion rates, the program was favorably received by participating social workers. Given these results, the distress screening process seemed to strike a balance at our institution between perceived benefit to social work practice and the burden of administration. Caregiver distress screening has now become routine practice for social workers serving patients with brain and solid tumors at our institution, and our department plans to expand the program to other diagnostic populations in the upcoming year. The insights we glean from continued screening will enable our institution to better tailor the psychosocial supports and interventions we provide to meet the unique needs and concerns of caregivers of our patients.

## Figures and Tables

**Table 1:** Frequency Table for Demographics.

Variable	*n*	%
Caregiver Gender		
Female	32	72.72
Male	12	27.27
Patient Diagnosis		
Brain Tumor (e.g., Medulloblastoma, Diffuse Intrinsic Pontine Glioma, Diffuse Midline Glioma, Atypical Teratoid Rhabdoid Tumor, Pilocytic Astrocytoma, High Grade Glioma, etc.)	39	88.63
Solid Tumor (e.g., Neuroblastoma, Malignant Peripheral Nerve Sheath Tumor, Nasopharyngeal Carcinoma, etc.)	5	11.36

**Table 2:** Frequency Table for Caregivers Whose Scores Averaged Moderate to Very Seriously Concerned.

CSS-CG Item	*n*	%
Worrying about the future and what lies ahead	27	61.36
Disruptions in work, school, or home life	25	56.81
Managing household finances	17	38.63
Feeling nervous or afraid	15	34.09
Feeling sad or depressed	15	34.09
Exercising and being physically active	14	31.81
Feeling lonely or isolated	13	29.54
The patient’s eating or nutrition	13	29.54
Keeping up with your healthcare needs	11	25
Changes in the patient’s mood or behavior	11	25

**Table 3:** Social Worker Survey Results.

Survey Item	SA (%)	A (%)	N (%)	D (%)	SD (%)
As a result of using the distress screener, my sessions are more focused.	50	33.30	16.70	0	0
As a result of using the distress screener, my sessions are more meaningful.	16.70	50	0	33.30	0
As a result of using the distress screener, my sessions are more intentional.	50	16.70	0	33.30	0
As a result of using the distress screener, my sessions are more collaborative.	33.30	16.70	33.30	16.70	0
As a result of using the distress screener, my sessions are more helpful to families.	50	16.70	33.30	0	0
As a result of using this tool, I am more able to identify caregiver concerns related to self-care.	50	16.70	33.30	0	0
As a result of using this tool, I am more able to identify caregiver concerns related to managing their child’s medical care and life at the hospital.	50	33.30	16.70	0	0
As a result of using this tool, I am more able to identify caregiver concerns related to managing life outside of the hospital.	50	50	0	0	0
As a result of using this tool, I am more able to identify caregiver concerns related to coping with difficult emotions.	50	33.30	0	16.70	0
Overall, using this tool helps me better identify caregiver needs.	66.70	16.70	16.70	0	0
In general, caregivers are willing to complete the questionnaire.	0	33.30	50	16.70	0
In general, caregivers find the process beneficial.	0	33.30	66.70	0	0
In general, using this screening tool positively impacts my working relationship with caregivers.	50	33.30	16.70	0	0
It is easy to incorporate this tool into my practice.	50	33.30	0	16.70	0
I have the time and resources I need to use this tool effectively.	33.30	33.30	16.7	16.70	0
The amount of time I spend on the distress screening process is reasonable.	50	33.30	0	16.70	0
Overall, using this tool is worth the effort because it helps improve my practice as a social worker.	33.30	50	0	16.70	0

Note: SA = Strong Agree, A = Agree, N = Neither Agree nor Disagree, D = Disagree, SD = Strongly Disagree.

## Data Availability

Data available upon request.
